# Fragility and basic process energies in vitrifying systems

**DOI:** 10.1038/srep08314

**Published:** 2015-02-09

**Authors:** Julio Cesar Martinez-Garcia, Sylwester J. Rzoska, Aleksandra Drozd-Rzoska, Szymon Starzonek, John C. Mauro

**Affiliations:** 1 Department of Chemistry and Biochemistry, University of Berne, Freiestrasse 3, Berne CH-3012, Switzerland; 2 Institute of High Pressure Physics Polish Academy of Sciences, ul. Sokołowska 27/39, 01-142 Warsaw, Poland; 3 Institute of Physics & ŚMCEBI, University of Silesia, ul. 75 Pułku Piechoty 1A, 41-500 Chorzów, Poland; 4 Science and Technology Division, Corning Incorporated, Corning, New York 14831, USA

## Abstract

The concept of ‘fragility’ constitutes a central point of the glass transition science serving as the ‘universal’ metric linking previtreous dynamics of qualitatively distinct systems. Finding the fundamental meaning of fragility is the ‘*condicio sine qua*’ for reaching the long expected conceptual breakthrough in this domain. This report shows that fragility is determined by the ratio between two fundamental process energies, viz. the activation enthalpy and activation energy. The reasoning, avoiding any underlying physical model, is supported by the experimental evidence ranging from low molecular weight liquids and polymers to plastic crystals and liquid crystals. All these lead to the new general scaling plot for dynamics of arbitrary glass former. The limited adequacy of broadly used so far semi-empirical relationships between fragility and the activation energy is shown. Results presented remain valid for an arbitrary complex system and collective phenomena if their dynamics is described by the general super-Arrhenius relation.

Glass transition constitutes one of grand challenges of condensed and soft matter physics as well as modern materials science^1^,^2^,^3^,^4^, where a long-expected fundamental breakthrough could lead to innovative implementations ranging from silicate glasses^5^ and plastics^6^ to pharmaceuticals^7^ and foods^8^. The ultimate progress in this area is also important for biotechnological^9^, geophysical^10^, metallurgical^11^ and electronic devices^12^ implementations. Notable is the fact that glass transition physics is considered as one of key references for collective phenomena science, aimed to discover properties emerging from complex correlations^13^.

Of particular interest of the glass transition research is the identification of universal features in the previtreous dynamic regime shared amongst a surprising variety of systems including low molecular weight liquids, polymers, liquids crystals, plastic crystals, colloids, metallic alloys, silicates, spin glasses, etc^2^,^3^,^4^,^5^. A key metric linking so distinct glass formers was introduced by Austen Angell^14^, basing on a master plot of log_10_*η*(*T*) and/or log_10_*τ*(*T*) versus *T_g_*/*T* where *η*(*T*) stands for viscosity, *τ*(*T*) for structural (*primary*) relaxation time and *T_g_* is the glass temperature^14^,^15^. This was possible due to the empirical normalization assumption for the glass transition temperature *η*(*T_g_*) = 10^13^ *Poise* and *τ*(*T_g_*) = 100 *s*. Subsequently, a metric describing the slope for *T* → *T_g_*, called ‘fragility’, was proposed^14^,^15^:
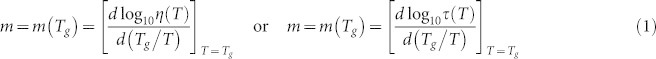
The fragility index *m* describes the degree of shifting from the basic Arrhenius dynamics to the super-Arrhenius (SA) one, described by the general form^2^:
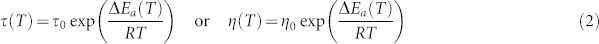
where *T* > *T_g_*, *R* denotes the gas constant and Δ*E_a_*(*T*) the apparent activation energy. The basic Arrhenius dependence is restored for Δ*E_a_*(*T*) = *E_a_* = *const*.

There are two general types of glass formation defined by the fragility metric: (**i**) ‘*fragile’* systems with highly SA dynamics (*m* > 50) and (**ii**) ‘*strong’* ones, with close-to-Arrhenius behavior (*m* < 30)^2^,^14^,^15^. The basic Arrhenius behavior *τ*(*T*) = *τ*_0_ exp(*E_a_*/*RT*) is associated with the minimal value of the fragility index and most often related to *m* = log_10_(*τ*(*T* = *T_g_*)) −log_10_ *τ*_0_ = 2 + 14 = 16, i.e. assuming for the prefactor *τ*_0_ = 10^−14^ *s* in the SA eq. (2)^2^,^15^. Notwithstanding, for silicate liquids extremely strong SA behavior with a minimal *m* = 14.93 was found^16^. Experimental estimations of the prefactor in the SA equation ranges from ~10^−11^ *s* to even ~10^−18^ *s*^2^,^17^, what indicates on the system-dependent minimal fragility.

Qualitative mapping of the previtrous increase of relaxation times or viscosity onto a single chart has led to the concept of fragility, becoming a focal point for research in glass transition physics^2^,^3^. The most important appeared as the link between two basic properties, viz. fragility and the activation energy^2^,^3^,^18^,^19^,^20^. One may claim that the ultimate explanation of this problem is the “*condicio sine qua*” for reaching the conceptual breakthrough in glass transition physics^2^,^3^. Surprisingly, despite decades of studies the situation is puzzling.

The first and broadly implemented up to now dependence^20^,^21^,^22^,^23^,^24^,^25^,^26^ was proposed by Boehmer et al.^15^ in 1993:
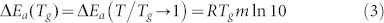
In 2004, Novikov and Sokolov^27^ proposed yet another relation, supported by experimental evidence for a set of glass forming liquids^27^,^28^:
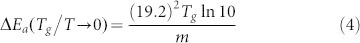


This report presents the critical discussion of eqs. (3) and (4) and shows that their validity is casual. Subsequently, it presents the lacking so far fundamental link between fragility and fundamental process energies, viz. the activation energy and the activation enthalpy. The new, ‘ultimate’, scaling relation linking fragility and the activation energy has been also derived. Analytic results are supported by the clear experimental evidence for a broad range of glass formers.

## Results

### The new insight into fragility of glass formers

In Refs. 29, 30 the new approach for the insight into dynamics of the previtreous domain, based solely on the SA eq. (2) and the metric describing relative changes of the apparent activation energy was introduced:
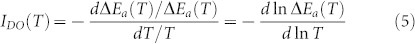
The analysis in Refs. 29, 30 was possible due to the innovative way of determining Δ*E_a_*(*T*), which avoids the biasing impact of generally unknown prefactor *τ*_0_ in the SA eq. (2). This model-free approach lead to a set of notable findings including the limited fundamental adequacy of the Vogel-Fulcher-Tamman (VFT)^31^,^32^,^33^ equation, identifying the role of local symmetry in glass formation and showing the ultimate way of “dynamic” estimation of the ideal glass transition temperature^29^,^30^.

Linking the SA eq. (2) and eq. (5) for the apparent activation energy temperature index one obtains:
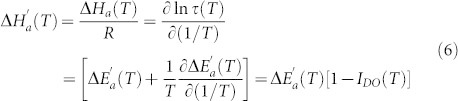
where the identity *d*(1/*T*) = (−(1/*T*)*dT*)(1/*T*) = −(*d* ln *T*)(1/*T*) was used, Δ*H_a_*(*T*) denotes the apparent activations enthalpy (see also Ref. 17 and Suppl. Info of Refs. 29, 30 where clear derivations of the relationship between Δ*E_a_*(*T*), Δ*H_a_*(*T*) and *d* ln *τ*(*T*)/*d*(1/*T*) are given), 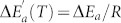. 

**Figure 1:**
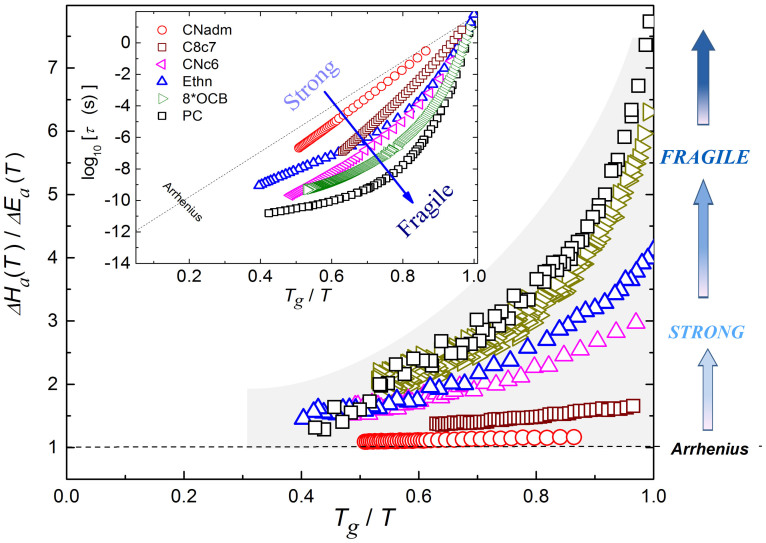
The evolution of the ratio between the apparent activation enthalpy and energy for selected glass forming systems. The inset shows the classic “Angell plot”^2^,^14^,^15^ for *τ*(*T*) experimental data, constituting the base for determining non-biased ratio of process energies in the main plot. For basic data see Table 1.

The above dependence directly yields:
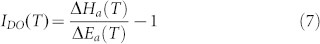


Figure 1 shows that eq. (7) can serve as the base for the new “universal scaling plot” for the previtreous dynamics of arbitrary glass former. It includes representatives from LMW, P, ODIC and LC categories^29^,^30^,^34^,^35^,^36^,^37^,^38^,^39^. Notable is the correlation with the classical ‘Angell’ plot^2^,^3^,^14^,^15^, presented in the inset for the same systems. For both plots the increase of curvature indicates the rise of fragility. However, in the main plot fragility it is directly determined by the ratio of fundamental process energies, namely:
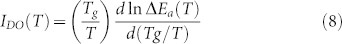
which lead to:
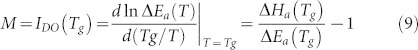
The onset of the Arrhenius behavior is associated with the negligible curvature and almost horizontal behavior for Δ*H_a_*(*T*)/Δ*E_a_*(*T*) ≪ 1 in Fig. 1.

The classical fragility index *m* (eq. (1)) is still explained as the “formal” parameter, namely the slope at the ‘Angell’ plot^2^,^14^,^15^. The new fragility parameter *M* = *I_DO_*(*T_g_*) gives directly value between two basic process energies, which are then key fundamental features determining the value of fragility. Using eqs. (1), (2) and (9) one obtains the link between the ‘classical’ (*m*) and new (*M*) fragility metrics:
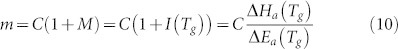
where the constant *C* = 2 − log_10_(*τ*_0_) = 13–18.

The relationship between *m* and *I_DO_*(*T_g_*) was indicated earlier by Hecksher et al.^40^, but without an explanation of the physical meaning of *I_DO_*(*T_g_*).

**Figure 2:**
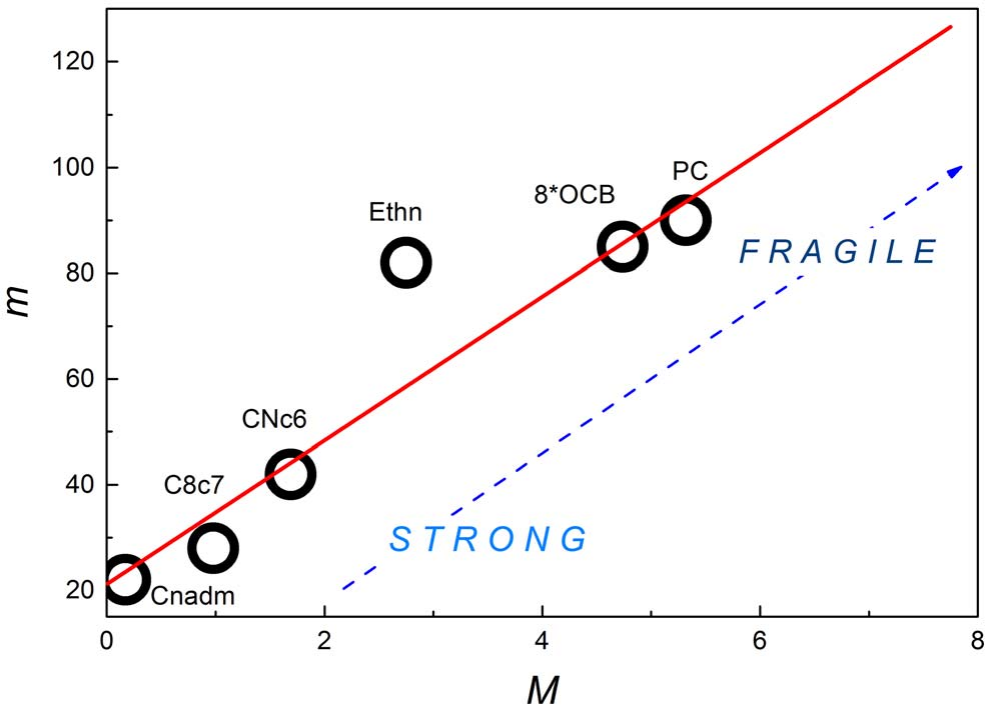
The experimental tests of the relationship between the ‘*classical*’ fragility metric *m* and the new metric *M* = Δ*H_a_*(*T_g_*)/Δ*E_a_*(*T_g_*) − 1. Results are for selected glass forming LMW, P, ODC and LC systems (see Table 1).

The experimental confirmation of the behavior predicted by eq. (10) is given in Figure 2. It is notable that eq. (10), showing also the link of *m* to basic process energies, indicates also the uncertainty introduced by the prefactor *τ*_0_ or *η*_0_ in SA eq. (2). The summary of characteristics for aforementioned experimental systems is given in Table 1.

**Figure 3:**
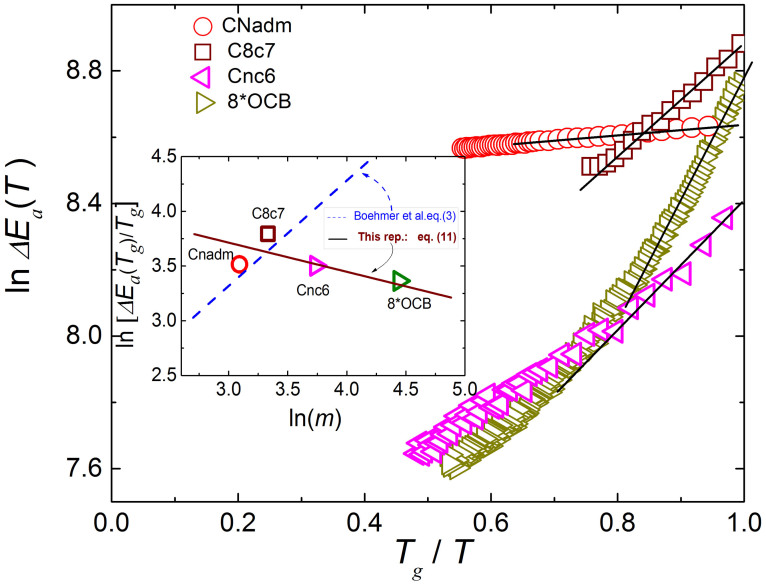
Degree of nonlinearity at “Arrhenius-type” plotted as ln(Δ*E_a_*(*T*)) vs. *T_g_*/*T* for representative glass formers. The figure indicates the lack of correlation between increasing curvature, coupled to fragility, and the value of Δ*E_a_*(*T_g_*). The clear disagreement with eq. (3) is stressed by the inset: the blue, dashed line is related to eq. (3) and the solid, black line is based on the MFR.

However, the most fundamental eqs. (1) and (2) directly indicate that the increasing SA behavior is associated with the rising nonlinearity at the “Arrhenius-type” plot ln(Δ*E_a_*(*T*)) vs. 1/*T*. Fig. 3 presents such plot, revealing the lack of a correlation between the increasing curvature of the apparent activation energy, coupled to rising fragility *m*, and the value of Δ*E*(*T_g_*). This is in clear disagreement with mentioned above basic prediction (eq. (3)). Moreover, the simply linearization based on eqs. (1) and (2) yields 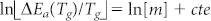, i.e. the linear function with the intercept at *cte* = ln[*R* ln 10] > 0 and the directional factor *b* = 1. Such prediction is anti-correlated with experimental data, as shown in the inset in Fig. 3 via the dashed line. Consequently, the used so far basic link between the activation energy and fragility Δ*E_a_*(*T_g_*) = *RT_g_m*ln10, i.e. (eq. (3))^2^,^14^,^16^,^20^,^21^,^22^,^23^,^24^,^25^,^26^, is inherently invalid.

**Figure 4:**
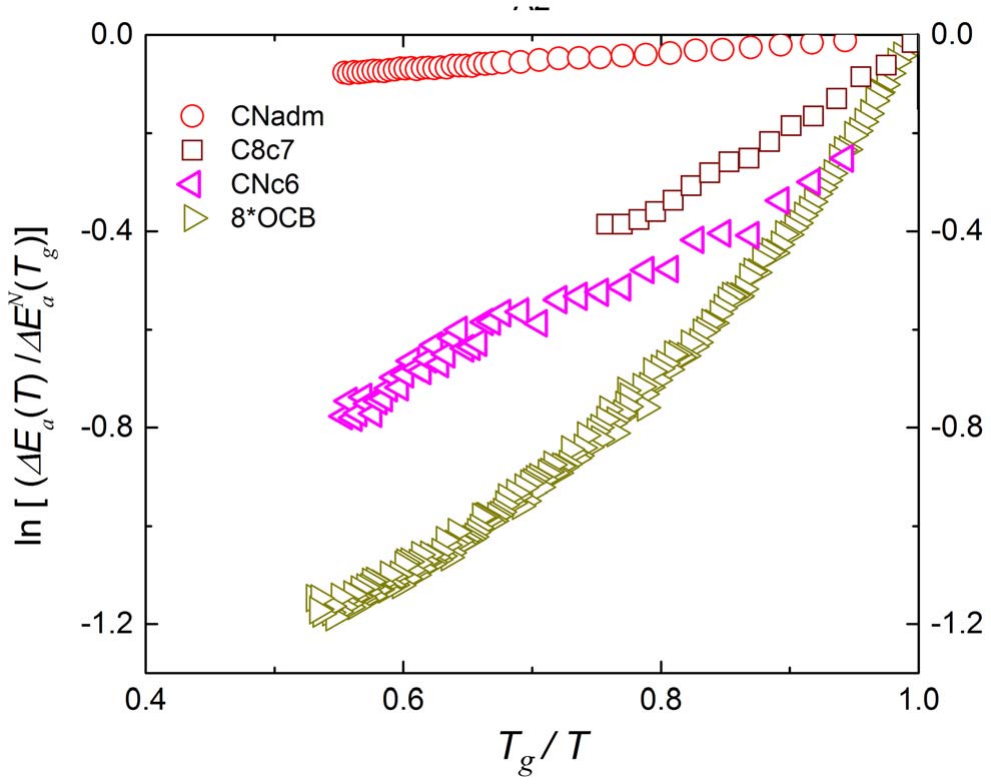
The “universal” scaling plot for activation energies in representative glass formers. The apparent activation energy is obtained by MFR^29^,^30^ procedure (see Methods) which is rescaled at *T_g_* using eq. (11). The figure shows the correlation between increasing curvature, indicating the rise of fragility, and Δ*E_a_*(*T_g_*). For basic data see also Table 1.

However, the simple analysis based solely on general eqs. (1) and (2) and eq. (9), derived above yields (see also Methods section):
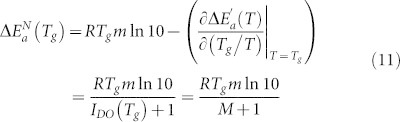
Figure 4 shows that the implementation of eq. (11) orders “chaotically scattered” curves in the main part of Fig. 3. It also leads to the superior agreement with experimental data given the inset in Fig. 3 (the solid line). Consequently, eq. (11) can be considered as the new ultimate link between the activation energy and fragility, valid for an arbitrary glass former.

**Figure 5:**
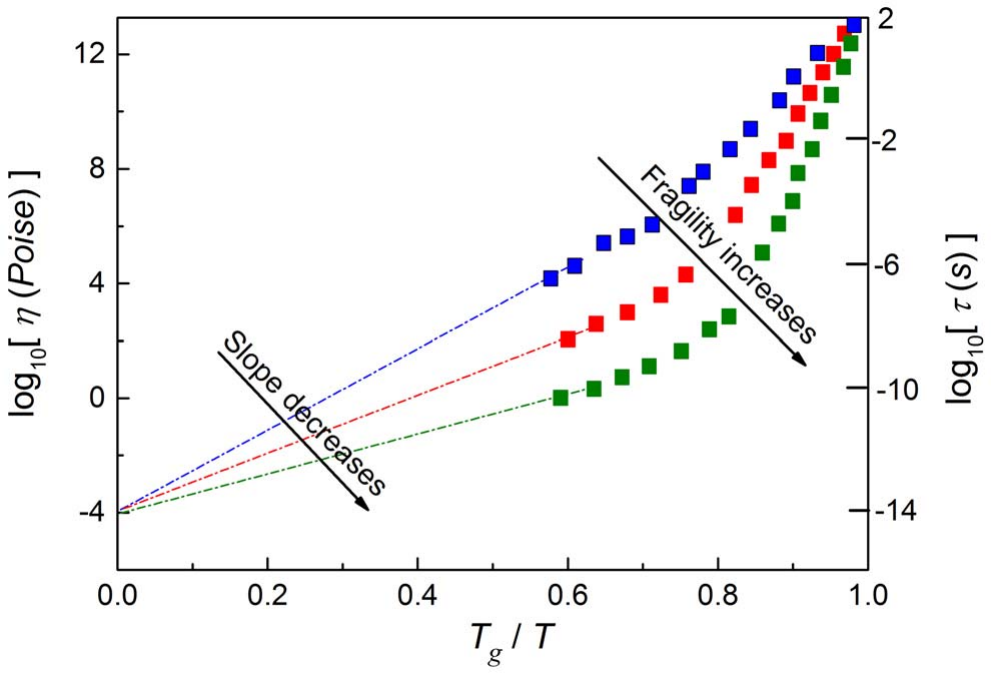
The schematic plot explaining the source of the hypothetical correlation between fragility *m* and the activation energy for the high temperature border case *T_g_*/*T* → 0. Fragility is related to the slope for *T_g_*/*T* → 1, in the ultraviscous, “low temperature”, dynamic domains (indicated by colored symbols). Lines are for the high temperature domain terminating at *T_g_*/*T* → 0. The plot recalls the basis of Novikov and Sokolov^27^,^28^ relationship linking fragility and activation energy in the high temperature domain (eq. (4)). The presumable “universal” high temperature (*T_g_*/*T* → 0) values of prefectors in SA eq. (2) are *η*_0_ = 10^−4^ *Poise* or *τ*_0_ = 10^−14^ *s*^2^.

Novikov and Sokolov^27^,^28^ proposed yet another relation linking fragility (determined for *T_g_*/*T* → 1) with the activation energy but this time taken for *T_g_*/*T* → 0, viz. eq. (4). For explaining its meaning, let's recall that in the low temperature domain (*T_g_*/*T* → 1) the increase of curvature and the slope in the ‘Angell’ plot^14^,^15^ is linked to increasing fragility *m*. For the same plot, log_10_ *η*(*T*) or log_10_ *τ*(*T*) vs. *T_g_*/*T*, in the high temperature domain the decreasing slope is related to increasing value of *m*. This behavior is illustrated in Figure 5, showing that Novikov and Sokolov^27^,^28^ relation Δ*E_a_*(*T_g_*/*T* → 0) ~ 1/*m* (eq. (4)) results from the construction of the ‘Angell’ plot. The underlying assumption of eq. (4) is also the “universal” value of the viscosity (log_10_ *η*_0_ = −4) or for relaxation time log_10_ *τ*_0_ = −14 for *T_g_*/*T* → 0. This border values are considered as hypothetical universal values of the prefactor in the SA eqs. (1).

However, the existence of such universal values of prefactors can be questioned, particularly when taking into account different categories of glass formers, as discussed above. All these indicate on the necessity of a supplementary analysis, related to: (i) the activation energy determined without the biasing impact of the prefactor and (ii) the impact of the qualitative differences between dynamic the high- and low- temperatures domains. The latter is particularly important, because eq. (4) suggests direct causal link between different dynamic domains.

Following the above discussion, as well as the evidence from Refs. 2, 29, 30, one can indicate following basic features of the ultraslowing/ultraviscous domain:

- There are no glass forming systems in the ultraviscous/ultrasowing domain where Δ*E_a_*(*T*) decreases on cooling, i.e. 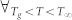 ∂Δ*E_a_*(*T*)/∂(1/*T*) ≥ 0 and *I_DO_*(*T*) ≥ 0.
- For extremely strong glass formers Δ*E_a_*(*T*) ~ Δ*H_a_*(*T*) for the whole low temperature dynamic domain. In the case of the Arrhenius behavior Δ*E_a_*(*T*) = Δ*H_a_*(*T*) = *const*.
- In the ultraviscous/ultraslowing domain always Δ*H_a_*(*T*) > Δ*E_a_*(*T*) and in the vicinity of *T_g_* even Δ*H_a_*(*T*) ≫ Δ*E_a_*(*T*). This is particularly evident for fragile ultraslowing and/or ultraviscous systems.

## Discussion

The fragility and the activation energy are the most fundamental characteristics of glass transition. One can expect that a conceptual progress in this challenging area of condensed matter physics needs the unequivocal relationship between these quantities. However, this basic problem appeared to be surprisingly difficult, viz. the title of the recent Ref. 42: “*The fragility and other properties of glass-forming liquids: Two decades of puzzling correlations*”.

The current report presents the first ever evidence of the unequivocal link between fragility and ratio of two basic process energies: the activation energy and the activation enthalpy in the low temperature ultraviscous/ultraslowing dynamic domain. It is worth recalling that the activation energy Δ*E_a_*(*T*) is associated with the energy barrier necessary to boost a process, i.e. a transition state's free energy (the energy barrier) minus the energy of substrate's. This report shows the direct link of fragility to the ratio of these energies: *m* = (2 − log_10_ *τ*_0_)(Δ*H_a_*(*T_g_*)/Δ*E_a_*(*T_g_*)), but affected by the uncertainty associated with the SA prefactor *τ*_0_ (or *η*_0_). This biasing impact can be avoided for the new fragility metric *M* = *I_DO_*(*T_g_*) = Δ*H_a_*(*T_g_*)/Δ*E_a_*(*T_g_*) − 1, ranging from *M* = 0 (the basic Arrhenius case) to *M* > 10 for strongly SA dynamics. The activation enthalpy can be easily calculated via 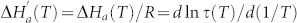 or 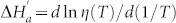 and the activation energy via the recently proposed model free route procedure (see Methods and Refs. 29, 30). This report shows that in the ultraviscous/ultraslowing domain always Δ*H_a_*(*T*) > Δ*E_a_*(*T*) and in the immediate vicinity of *T_g_* even Δ*H_a_*(*T*) ≫ Δ*E_a_*(*T*). We emphasize this issue, since in a number of research reports the erroneous assumption that 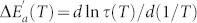 near the glass transition has been used^20^,^21^,^22^,^23^,^43^,^44^,^45^,^46^,^47^,^48^,^49^.

One of the most attracting questions regarding fragility is its maximal value. In Ref. 50 value *m* ≈ 175 was indicated as the maximal possible fragility, what is related to *M* ≈ 10. However, earlier *m* ≈ 214 was noted as the indicator of the most SA dynamics^51^. Basing on this report, recalling the basic Adam-Gibbs (AG) theory^2^,^52^ and Refs. 29, 30 the following general dependence for the apparent activation energy temperature index can be obtained (see Methods section):
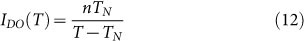
The latter dependence and eq. (9) yields:
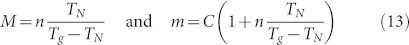
For example, for glass formers with rod-like molecules and the clear uniaxial, orientational symmetry *n* ≈ 1.6 and *T_g_* − *T_N_* ≈ 10 with *T_N_* ≈ 300 *K* (see Suppl. Info to Ref. 30) one obtains *m* ≈ 280!

**Figure 6:**
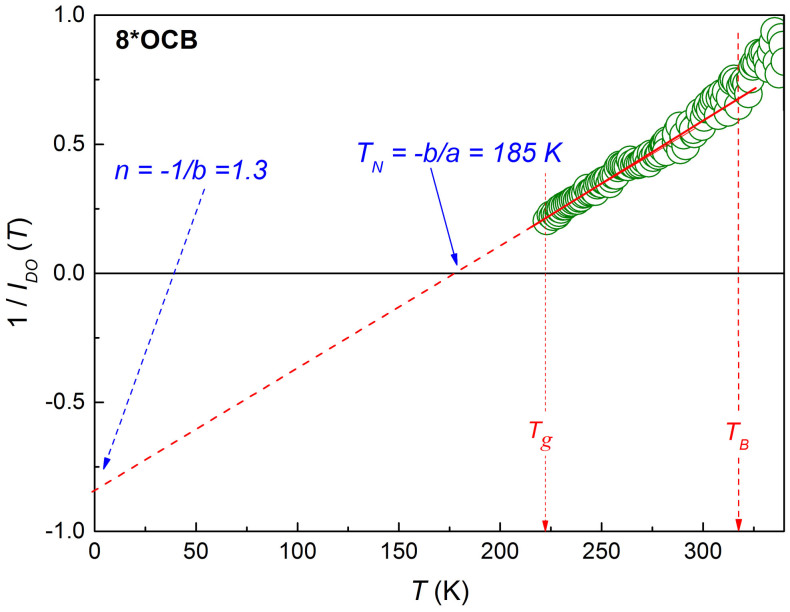
The temperature scaling behavior of the reciprocal of the apparent activation temperature index in glass forming liquid crystalline *n*-octylocycyanobiphenyl (8*OCB). The ultraviscous domain extends between *T_g_* < *T*(≈ 100 *K*) < *T_B_*, where the latter denotes the dynamic crossover temperature^2^. Locations of the singular temperature *T_N_* and the extrapolation down to *T* = 0 as well as the method of calculating the experimental dependence 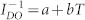 are shown^29^,^30^.

Following the given report and Refs. 29, 30 one can postulate that the transformation of *τ*(*T*) or *η*(*T*) experimental data to *I_DO_*(*T*) representation can yield all basic characteristics of previtreous dynamics, basing solely on inherently unambiguous linear regression fit, namely: (i) the local symmetry related parameter 
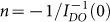
, (ii) the extrapolated singular temperature for which 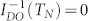 and (iii) the fragility metric *M* = *I_DO_*(*T_g_*), linked to basic process energies. Knowing (*T_N_*,*M*,*n*) (see Fig. 6 in Methods section) and the fragility *m* (from the “Angell” plot) the unambiguous estimation of *τ*_0_ or *η*_0_ prefactors is also possible.

One of still mysterious features of the glass transition are different distances between the glass temperature (*T_g_*) and the extrapolated Kauzmann temperature (*T_K_*) in various glass formers^2^,^53^. Following the finding the *T_N_* = *T_K_* (see Ref. 30 and Methods section) and eq. (13) one obtains *M*/*n* = *T_N_*/(*T_g_* − *T_N_*) = *M*/*n*, i.e. the relative distance between *T_K_*( = *T_N_*) and *T_g_* depends solely on the fragility (*M*) and symmetry related (*n*). It is notable that eq. (13) makes it possible also unequivocal calculation the *τ*_0_ for the SA eq. (2), avoiding any “ersatz equations”, like the VFT^2^ one used so far.

Worth noting is the relationship of the new fragility parameter *M* to the one introduced by Doremus^54^, who applied the empirical finding that at high temperature (HT) the activation energy low (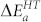, originally denoted as *Q_L_* is low) whereas at low temperatures prior to the vitrification its value is high (*Q_H_*, 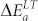). This lead to the Doremus fragility metric 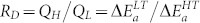 with *R_D_* < 2 for “strong” glass formers and *R_D_* > 2 for “fragile” ones^2^,^54^. In the Doremus model *Q_H_* = *H_d_* + *H_m_*, where *H_d_* is associated with enthalpy of formation of broken bonds and *H_m_* is responsible for motions. At high temperature *H_m_* dominates and *Q_L_* = *H_m_*. Such picture results from the fact that Doremus introduced the model for ultraviscous silica, but its extension to other glass formers can be explained by the formations of bond-ordering local structures or heterogeneities. This led Doremus to the double-exponential function for description of the viscous flow, at least in silicates^54^. This report correlates with some fundamental of Doremus model^54^, although the analysis avoids any model assumptions, basing solely on the general Super-Arrhenius equation (eq. (2)).

It is also notable that results of the given report and empirical findings in Refs. 29, 30 lead to surprisingly simple form of relative changes of process energies in the ultraviscous/ultraslowing domain Δ*E_a_*(*T*)/[Δ*H_a_*(*T*) − Δ*E_a_*(*T*)] = *a* + *bT* with *a* ≠ 0 and *b* ≠ 0.

Concluding, this reports presents the link between fragility and basic process energies, in ultraviscous/ultraslowing glass forming materials lacking so far. However, results presented above are also significant for complex systems and collective phenomena if their dynamics is described by the general super-Arrhenius relation.

## Methods

### The analysis of experimental data and the model-free route (MFR) method

The “*model-free*” route procedure introduced in Refs. 29, 30 is a novel approach for getting insight into previtreous dynamics based on the transformation of basic structural relaxation time (*τ*(*T*)) or viscosity (*η*(*T*)) experimental data to apparent activation energy temperature index form, the magnitude first proposed for glass formers by Dyre and Olsen (DO)^40^ via:
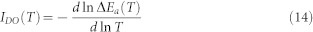
The apparent activation energy 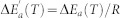 is determined from *τ*(*T*) and *η*(*T*) experimental data via the solution of the differential equation resulting from the general SA eq. (2)^29^,^30^:
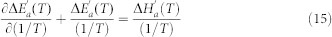
where the apparent activation enthalpy is given by 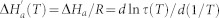
29.

This way of determining 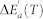 was only recently introduced in Refs. 29, 30. Previously, the apparent activation energy was calculated from the SA eq.(2) as Δ*E_a_*(*T*) = *RT* ln (*τ*(*T*)/*τ*_0_)^2^,^40^,^41^. and then it was inherently biased by the generally unknown estimation of *τ*_0_ prefactor^2^. In practice, a “universal” value of *τ*_0_ = 10^−14^ *s* was most commonly assumed^2^,^40^,^41^. An inherent advantage of the MFR approach for determining Δ*E_a_*(*T*) and *I_DO_*(*T*) includes also the application of a numerical filtering procedure based on Savitzky-Golay principle^29^,^30^.

In Refs. 29, 30 the MFR have been implemented for a set of 55 glass forming systems, ranging from low molecular weight liquids (LMW) and polymers (P) to liquid crystal (LC), plastic crystal (ODIC) and spin glasses (SGL), in the previtreous domain. The analysis revealed a surprisingly simple pattern for the previtreous dynamics: 1/*I_DO_*(*T*) = *aT* + *b*, with *a* ≠ 0 and *b* ≠ 0 for all mentioned data sets. This led to the derivation of the new generalized configurational entropy equation *S_c_*(*T*) = *S*_0_(1 − (*T_N_*/*T*))*^n^*, where the power exponent is determined as 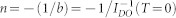 and *T_N_* is the singular temperature estimated via *I_DO_*(*T* = *T_N_*)^−1^ = 0^29^,^30^. Recently, basing on the MFR, the clear coincidence between the ideal glass (Kauzmann) temperature *T_K_* and *T_N_*, i.e. *T_K_* = *T_N_*, was found^30^. The analysis carried out in Refs. 29, 30 revealed that the parameter ranges between 0.18 < *n* < 1.53, where the lower limit is for systems with the clear positional symmetry (PS) and the higher one for systems with clear orientational, uniaxial symmetry (OS). The dynamics of PS and OS glass formers is relatively well portrayed by the critical-like equation^29^,^38^. The third characteristic case is for systems where *n* = 1 (no-symmetry). Only in this case the application of the popular Vogel-Fulcher-Tammann (VFT)^2^ equation is suitable^29^,^30^. Consequently, the fundamental justification of the VFT relation is limited to a small group of glass formers and otherwise (i.e. for *n* ≠ 1) it can be considered solely as an effective fitting tool.

The example of analysis employing the MFR analysis, based on transformed *τ*(*T*) experimental data in supercooled liquid crystalline *n*-octyloxycyanobiphenyl (8*OCB), is shown in Fig. 6. The way of determining the basic parameters is indicated.

Values of primary relaxation times *τ*(*T*) were determined as the reciprocal of the peak frequency of *ε*″(*f*) loss curve, obtained from broad band dielectric spectroscopy measurement (see Refs. 29, 30).

### Derivation of the general form for the activation energy temperature index (eq. (12)

Recalling the Adam-Gibbs theory for glass transtion^2^,^52^, the apparent activation index can be written as^29^,^30^:
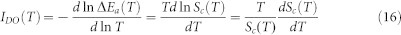
Substituting the new generalized configurational entropy (Ref. 29) *S_c_*(*T*) = *S*_0_(1 − (*T_N_*/*T*))*^n^*, one obtains:
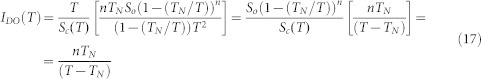


The above dependence make it possible to identify the impact of the entropic contribution in the anomalous behavior of the activation entropy temperature index.

Alternatively eq. (12) can be derived recalling the experimental finding that 1/*I_DO_*(*T*) = *aT* + *b*, where *b* = (−1/*n*) ≠ 0 and *a* = (1/*nT_N_*) ≠ 0 (the coefficient 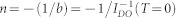 and the singular temperature *I_DO_*(*T* = *T_N_*)^−1^ = 0)^29^,^30^, one obtains Δ*H_a_*(*T*)/Δ*E_a_*(*T*) = [*T_N_*(*n* − 1) + *T*]/(*T* − *T_N_*) and then *I_DO_*(*T*) = *nT_N_*/(*T* − *T_N_*).

## Author Contributions

J.C.M.G., S.J.R. and A.D.R. wrote the main manuscript, J.C.M.G. prepared figures, S.z.S. worked on data analysis, J.C.M. finally shaped the form of the manuscript. Authors' related experimental results are associated with measurements carried out J.C.M.G., S.J.R. and A.D.R. New conceptions proposed in the paper are proposed mainly by J.C.M.G. and S.J.R. but finally influenced by A.D.R. and J.C.M.

## Figures and Tables

**Table 1:** The collection of basic “dynamic” characteristics parameters for the tested glass forming materials, Glass forming systems analyzed in the given research report. Footnotes close to short names of compounds are for references recalling experimental data sources. Numbers in parentheses “( )” denote the value of the fitting errors. The parameters n and T_N_ are for the “symmetry-related” exponent n and the singular temperature T_N_ determined via the linear regression fit at 1/I_DO_(T) plot. The glass transition temperature was estimated using the empirical condition τ(T_g_) = 100 s. The Angell fragility index and the new metric parameters are denoted by m and M respectively. The last column (R) gives the range (T_g_-T_end_) of tested experimental data

Sym.	System	Full name	*T_g_/K*	*T_N_/K*	*n*	*m*	*M*	*R/K*
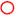	CNadm^34^	Cyanoadamantane (ODIC)	154	143 (3)	0.14 (0.06)	23	0.17 (0.08)	183–298
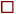	C8c7^35^	Cycloheptanol(57%) + Cyclooctanol(43%) (ODIC)	149	119 (2)	0.40 (0.08)	28	0.98 (0.09)	155–233
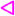	CNc6^36^	Cyanocyclohexane (ODIC)	134	120 (2)	0.14 (0.08)	48	1.69 (0.07)	138–277
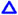	Ethn^37^	Ethanol (LMW)	99	72 (2)	1.23 (0.09)	52	2.75 (0.08)	96–250
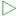	8*OCB^38^	Isooctylcyanobiphenyl (LC)	221	190 (3)	1.51 (0.08)	85	4.74 (0.06)	224–413
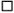	PC^39^	Propylene carbonate (LMW)	157	132 (2)	1.13 (0.09)	90	5.32 (0.06)	159–370
